# Supplementary material for: Meta-Analysis and Gene Set Enrichment Relative to ER Status Reveal Elevated Activity of MYC and E2F in the “Basal” Breast Cancer Subgroup
Source: PLoS One. 2009 Mar 9;4(3):e4710. doi: 10.1371/journal.pone.0004710 (PMC2650420; doi:10.1371/journal.pone.0004710)
Supplement: Dataset S1 — GSEA results from first screen. The file contains a zipped archive of the HTML results of the second GSEA screen. Reports for enriched or depleted datasets may be accessed from the “index.html” link within the my_analysis.GseaPreranked.1219975950625.rpt.zip ZIP archive. Enrichment plots are available for each of the 50 most enriched and depleted gene sets. (13.32 MB ZIP) [file pone.0004710.s001.zip › my_analysis.GseaPreranked.1219975950625.rpt.zip!//CHR16P11.html]

Details for gene set CHR16P11[GSEA]

|  || Dataset | zSco.ERneg.to.ERpos\_collapsed |
| Phenotype | NoPhenotypeAvailable |
| Upregulated in class | na\_neg |
| GeneSet | CHR16P11 |
| Enrichment Score (ES) | -0.44811004 |
| Normalized Enrichment Score (NES) | -1.8448906 |
| Nominal p-value | 0.0 |
| FDR q-value | 0.027544204 |
| FWER p-Value | 0.629 |
Table: GSEA Results Summary

  

Fig 1: Enrichment plot: CHR16P11      
 Profile of the Running ES Score & Positions of GeneSet Members on the Rank Ordered List

  

| PROBE | GENE SYMBOL | GENE\_TITLE | RANK IN GENE LIST | RANK METRIC SCORE | RUNNING ES | CORE ENRICHMENT || 1 | LOC440366 |  |  | 1953 | 2.568 | -0.1189 | No |
| 2 | CORO1A |  |  | 2626 | 2.131 | -0.1460 | No |
| 3 | NFATC2IP |  |  | 2783 | 2.050 | -0.1351 | No |
| 4 | COX6A2 |  |  | 2905 | 1.983 | -0.1223 | No |
| 5 | MYLPF |  |  | 2958 | 1.955 | -0.1046 | No |
| 6 | QPRT |  |  | 3177 | 1.850 | -0.1006 | No |
| 7 | ASPHD1 |  |  | 3535 | 1.692 | -0.1089 | No |
| 8 | ZNF267 |  |  | 3725 | 1.614 | -0.1053 | No |
| 9 | IL21R |  |  | 3833 | 1.578 | -0.0959 | No |
| 10 | SRCAP |  |  | 4939 | 1.175 | -0.1663 | No |
| 11 | SPN |  |  | 4943 | 1.175 | -0.1535 | No |
| 12 | CD19 |  |  | 5001 | 1.156 | -0.1450 | No |
| 13 | ERAF |  |  | 5274 | 1.057 | -0.1539 | No |
| 14 | ATXN2L |  |  | 5968 | 0.825 | -0.1970 | No |
| 15 | ITGAX |  |  | 6751 | 0.572 | -0.2496 | No |
| 16 | ITGAM |  |  | 6986 | 0.493 | -0.2618 | No |
| 17 | SULT1A3 |  |  | 7064 | 0.463 | -0.2625 | No |
| 18 | RNF40 |  |  | 7592 | 0.272 | -0.2993 | No |
| 19 | TBX6 |  |  | 7723 | 0.223 | -0.3066 | No |
| 20 | SETD1A |  |  | 7724 | 0.222 | -0.3041 | No |
| 21 | ZNF646 |  |  | 7743 | 0.216 | -0.3031 | No |
| 22 | TAOK2 |  |  | 8343 | -0.013 | -0.3481 | No |
| 23 | FUS |  |  | 8367 | -0.024 | -0.3496 | No |
| 24 | VKORC1 |  |  | 8651 | -0.153 | -0.3692 | No |
| 25 | PRR14 |  |  | 8667 | -0.158 | -0.3686 | No |
| 26 | KIF22 |  |  | 8725 | -0.180 | -0.3709 | No |
| 27 | C16ORF67 |  |  | 9209 | -0.409 | -0.4028 | No |
| 28 | APOB48R |  |  | 9286 | -0.450 | -0.4036 | No |
| 29 | TUFM |  |  | 9339 | -0.477 | -0.4023 | No |
| 30 | MAZ |  |  | 9415 | -0.521 | -0.4022 | No |
| 31 | IL4R |  |  | 9609 | -0.623 | -0.4098 | No |
| 32 | ZNF747 |  |  | 9815 | -0.734 | -0.4172 | No |
| 33 | SH2B1 |  |  | 9838 | -0.747 | -0.4106 | No |
| 34 | CCDC101 |  |  | 9924 | -0.795 | -0.4082 | No |
| 35 | TBC1D10B |  |  | 10137 | -0.925 | -0.4140 | No |
| 36 | CTF1 |  |  | 10591 | -1.235 | -0.4345 | Yes |
| 37 | XTP3TPA |  |  | 10592 | -1.236 | -0.4208 | Yes |
| 38 | BCL7C |  |  | 10616 | -1.256 | -0.4087 | Yes |
| 39 | FBS1 |  |  | 10817 | -1.402 | -0.4082 | Yes |
| 40 | MYST1 |  |  | 10922 | -1.499 | -0.3995 | Yes |
| 41 | KCTD13 |  |  | 10990 | -1.559 | -0.3873 | Yes |
| 42 | DOC2A |  |  | 11239 | -1.778 | -0.3864 | Yes |
| 43 | C16ORF53 |  |  | 11277 | -1.809 | -0.3692 | Yes |
| 44 | ZNF768 |  |  | 11572 | -2.104 | -0.3681 | Yes |
| 45 | TGFB1I1 |  |  | 11729 | -2.249 | -0.3550 | Yes |
| 46 | MAPK3 |  |  | 11838 | -2.358 | -0.3371 | Yes |
| 47 | ZNF764 |  |  | 11879 | -2.418 | -0.3134 | Yes |
| 48 | CD2BP2 |  |  | 11951 | -2.496 | -0.2912 | Yes |
| 49 | STX4 |  |  | 11991 | -2.547 | -0.2660 | Yes |
| 50 | PHKG2 |  |  | 12030 | -2.600 | -0.2401 | Yes |
| 51 | ZNF629 |  |  | 12056 | -2.649 | -0.2127 | Yes |
| 52 | ZNF668 |  |  | 12223 | -2.876 | -0.1935 | Yes |
| 53 | ZNF688 |  |  | 12235 | -2.894 | -0.1623 | Yes |
| 54 | PRSS8 |  |  | 12276 | -2.934 | -0.1329 | Yes |
| 55 | SEPHS2 |  |  | 12372 | -3.056 | -0.1063 | Yes |
| 56 | GDPD3 |  |  | 12415 | -3.125 | -0.0749 | Yes |
| 57 | C16ORF58 |  |  | 12524 | -3.298 | -0.0466 | Yes |
| 58 | MVP |  |  | 13004 | -4.466 | -0.0334 | Yes |
| 59 | TMEM142C |  |  | 13169 | -5.172 | 0.0114 | Yes |
Table: GSEA details [plain text format]

  

Fig 2: CHR16P11: Random ES distribution      
 Gene set null distribution of ES for **CHR16P11**
